# Exploring health system challenges and gaps for crisis response in Ethiopia: a scoping review of publications and reports from 2020-2024

**DOI:** 10.1186/s12913-025-13084-y

**Published:** 2025-07-04

**Authors:** Asmamaw Atnafu, Getachew Teshale, Endalkachew Dellie, Young Su Park

**Affiliations:** 1 Department of Health Systems and Policy, Institute of Public Health, College of Medicine and Health Sciences, University of Gondar, Gondar, Ethiopia; 2 Department of the History of Medicine and Medical Humanities, College of Medicine, Seoul National University, Seoul, Republic of Korea

**Keywords:** Health systems, Crises response, Ethiopian, Public health emergencies

## Abstract

**Background:**

Ethiopia’s health system has faced significant challenges due to COVID-19, natural disasters, and conflicts, disrupting healthcare delivery. This scoping review examined health system gaps and responses during crises, aiming to recommend strategies for resilience.

**Method:**

We followed a systematic scoping review approach using the five-step methodology: defining the research question, identifying relevant literature, selecting studies, charting the data, and summarizing/analyzing results. The review was guided by PRISMA-ScR and used the Population, Concept, and Context (PCC) framework. We included all English-language studies published between January 2000 and December 2024 addressing challenges or responses of the Ethiopian health system during crises, regardless of study design. PubMed, SCOPUS, Google Scholar, and Google were searched using MeSH terms/keywords. After title, abstract, and full-text screening, 44 articles were included for final analysis. Data were synthesized using descriptive analytical methods and narrative synthesis to summarize and interpret findings.

**Results:**

Findings revealed crises severely impacted health services due to shortages of medical supplies, workforce, and infrastructure. Demand surges, transport restrictions, border closures, and financial constraints exacerbated these gaps. Additional challenges included poor data availability, high staff turnover, lack of emergency communication plans, and insufficient funding.

**Conclusion and recommendations:**

The COVID-19 pandemic and the northern Ethiopian conflict notably disrupted routine services, infrastructure, and health information systems.

To build resilience, the study recommends: (1) developing scalable emergency plans for essential services, (2) strengthening primary healthcare and community-based systems, (3) maintaining medical stockpiles, and (4) providing crisis-specific training for health workers. These measures aim to enhance preparedness and sustainability in Ethiopia’s health system amid future shocks.

**Supplementary Information:**

The online version contains supplementary material available at 10.1186/s12913-025-13084-y.

## Introduction

A crisis, in general, is a situation of instability or danger that significantly disrupts normal operations and requires immediate attention and action [1, 2]. In the context of a health system, a crisis can arise from various sources like pandemics or epidemics, natural disasters, political or social unrest, economic crisis and technological failures [3–5]. Both natural and man-made crisis leads to shortage of medical supplies, staffing challenges, and service disruptions. It also compromises quality of care, and evidence-based practices [6–9].

Within the previous decade, Ethiopia has challenged by droughts, flooding, locust infestations, armed conflicts, political instability, internal displacement and economic crisis [10–13]. These crises; especially the armed conflict in the northern and the social unrest in different parts of the country leads to reallocation of health resources to emergency response reducing the availability of routine healthcare services, damage health facilities and their infrastructures [14, 15]. The synergetic impact of these crises also increases in disease incidence and mortality. It also disrupted services, compromised quality of care, poor living conditions, and increased vulnerability of the affected population [13].

A study conducted in eastern Tigray showed that 83% of the health system and 55.8% of the health facilities were damaged by the conflict between Tigray and Federal government of Ethiopia [16]. In the same way; a qualitative study done in Amhara region also showed that the conflict caused distraction of health facilities and infrastructure, fleeing of health workers, interruption of healthcare services. Insecurity and lack of transportation due to this conflict also leads to shortage of medication, lifelong disabilities, post-traumatic stress disorders [14].

Another qualitative study also showed that the conflict happened in the northern Ethiopia results partial or complete shutdown of medical institutions, breakdown of the health information system and outbreaks of communicable diseases [17]. Similarly, studies done in Oromia region showed that about 8%, 12% and 14% of hospitals, health centers and health posts have been damaged, looted and/or completely destroyed due to the ongoing armed conflict in Oromia. This problem is exacerbated by severe drought as driven by climate change [15].

A study done on COVID-19 response challenges in Ethiopia also showed that the pandemic hindered the capacity to avail full packages of personal protective equipment in health facilities and intensive care capacity. The pandemic also affected the delivery of maternal, child and new-born services, prevention, and treatment of childhood illness, including immunization services [18].

Getting health impact data to design appropriate intervention and build sustainable and resilient health systems is difficult as there are few baseline and post crisis data [19]. Lack of robust local scientific evidence at a time of pandemic and epidemic occurrence is also the major challenge to design and implement control measures which is adaptable to local context and the outbreak patterns [20].

Several countries have demonstrated effective strategies for maintaining resilient health systems during crises. The Cuba surge capacity model [21] and the Thailand’s healthcare financing resilience strategy [22] are some of the effective strategies that Ethiopia can adopt and implement during crisis.

The World Health Organization (WHO) identifies six essential building blocks of a health system: service delivery, health workforce, health information systems, access to essential medicines, healthcare financing, and leadership/governance (WHO, 2007). These components work interdependently to ensure effective, equitable, and efficient healthcare [23].

In Ethiopia, there are few studies and unpublished reports on the impact of crisis on the health systems. However, these studies were focused on specific health system building blocks and for specific crisis. Compiled studies on health system challenges and evidence gaps during crisis are very crucial to provide a balanced, evidence-informed foundation for policies and to design strategies for sustainable and resilient health systems. Thus, this scoping review synthesizing fragmented evidences to identify health system challenges and gaps during crisis and suggests strategies to build sustainable and resilient health systems. The findings from this scoping review offer critical insights for policymakers, intervention/project designers, and researchers working to strengthen health systems in crisis-prone settings like Ethiopia. Policy makers may use the findings to prioritize health system resilience, and strengthen governance and coordination. Project designers and researchers may use the findings as a reference, problem prioritization and gap identification for intervention.

## Methods

### Study design

This study is a systematic scoping review of studies and reports carried out using qualitative, quantitative or mixed methods on health system challenges and response to crisis in Ethiopia. We have used a scoping review to capture the broad nature of the research question and the range of health system challenges and responses implemented across the health system building blocks. Our review follows the five-steps of the scoping review methodology detailed in Arksey and O’Malley’s framework, which includes: (1) defining the research question; (2) identifying relevant literature; (3) selecting studies; (4) charting the data; and (5) summarizing and analyzing the results [24].

### Identifying research questions

The following questions guided this scoping review: (1) what are the current challenges for the health system in responding to the crisis in Ethiopia? (2) What are the evidence gaps related to the crisis management challenges of the health system in Ethiopia that need to be further investigated? (3) How has Ethiopia’s health system responded to the crisis? To address these questions, we have adopted the Population, Concept and Context (PCC) framework developed by the Joanna Briggs Institute, which is described in Table 1.

**Table 1:** Population concept context (PCC) framework for defining the eligibility of the study main research questions

Criteria	Elements	Descriptions
P-Population	Health system entities, professionals, and stakeholders in Ethiopia	Includes healthcare providers, health administrators, policymakers, and organizations both the public and private, involved in health system response and crisis management at all administrative levels (national, regional, zonal, woreda, kebele) within Ethiopia
C-Concept	Health system challenges and crisis response, mapped to the WHO six health system building blocks	Focuses on identifying and synthesizing evidence regarding challenges, gaps, and responses related to the six WHO health system building blocks: service delivery, health workforce, health information systems, access to essential medicines, financing, and leadership/governance
C-Context	Crisis situations in Ethiopia, specifically from 2020 to 2024	Encompasses the context of public health emergencies and crises (such as natural disasters, political or social unrest, economic crisis and technological failures, COVID-19 pandemic, and related events) occurring in Ethiopia between 2020 and 2024, with an emphasis on national and subnational health system responses
Inclusions criteria	All studies, reports and grey literature published in English between January 2000 and December 2024, irrespective of the study design.	
Exclusion criteria	Studies for which the full-text was unavailable, expert’s opinions, debates, letters to the editor and studies and reports done on crisis but that do not specifically address the health system challenges or responses during crisis.	

### Eligibility criteria

All studies related to gaps or responses focusing on the six-health system building blocks (service delivery, health workforce, health information systems, access to essential medicines, healthcare financing, leadership and governance) during crisis in Ethiopia were included in the review. The inclusion criteria were articles, reports and grey literatures, such as reports by government institutional or Non-Governmental Organizations (NGO) (e.g., WHO, UNICEF, Ethiopian Ministry of Health), government and policy documents, conference papers, preprints, and dissertations published in English between January 2000 and December 2024, regardless of the study design. The exclusion criteria were studies for which the full-text was unavailable, expert’s opinions, conference papers, debates, letters to the editor and studies and reports done on crisis but that do not specifically address health system challenges or responses during crisis (Table 1). We have presented this paper as a narrative review, following some components of the Priority Reporting on Systemic Reviews and Metrics (PRISMA) guideline for scoping reviews (Supplementary file 1).

### Search strategy and information sources

A comprehensive search strategy has been used to identify relevant scientific studies and documents. The combination of MeSH terms and keywords related to health system building blocks, crisis, and Ethiopia were searched in both PubMed and SCOPUS. We chose PubMed and Scopus because they both comprehensively cover biomedical and interdisciplinary literature relevant to health systems. Additional literatures were identified through searching in Google scholar and Google. Furthermore, the ministry of health policy documents, annual performance reports, evaluation reports and other guidelines were incorporated. The database search strategy was designed to capture the most pertinent studies and included index terms for the three major concepts: (1) health system or healthcare system, (2) crisis, armed conflict, disaster, or violence and (3) Ethiopia, along with the respective synonyms.

The Boolean operators ‘OR’ and ‘AND’ were applied, as well as the proximity operator, to specify the allowed distance between the corresponding free-text words [25]. A detailed description of the search strategy used in these databases is provided in Table 2.

**Table 2:** Searching strategy in the study of health system challenges and evidence gaps for crisis response in Ethiopia

Database	Search terms	Result
PubMed (Title/Abstract)	“Health system“[Mesh] OR “Healthcare System “[Mesh] OR"Health System Building Blocks“[Mesh] OR “Service delivery“[Mesh] OR “Health Information Systems“[Mesh] OR “Access to Essential Medicines“[Mesh] OR “Health Workforce“[Mesh] OR “Leadership and Governance“[Mesh] OR “healthcare financing“[Mesh] AND “crisis“[Mesh] OR “emergency“[Mesh] OR “disaster” [Mesh] OR “armed conflict” [Mesh] OR “violence“[Mesh] AND “Ethiopia” [Mesh] OR “horn of Africa” OR “Federal Democratic Republic of Ethiopia” [Mesh]	300
SCOPUS (Article title/ Abstract/ keywords)	TITLE-ABS-KEY(“Health system” OR “Healthcare System” OR “Health System Building Blocks” OR “Service delivery” OR “Health Information Systems” OR “Access to Essential Medicines” OR “Health Workforce” OR “Leadership and Governance” OR “healthcare financing”) AND TITLE-ABS-KEY(“crisis” OR “emergency” OR “disaster” OR “armed conflict” OR “violence”) AND TITLE-ABS-KEY(“Ethiopia” OR “horn of Africa” OR “Federal Democratic Republic of Ethiopia”)	347
Google (100 hit)	(“health system” OR “healthcare system” OR “service delivery” OR “health information systems” OR “access to essential medicines” OR “health workforce” OR “leadership and governance” OR “healthcare financing”) AND (“crisis” OR “emergency” OR “disaster” OR “armed conflict” OR “violence”) AND (“Ethiopia” OR “Federal Democratic Republic of Ethiopia”)	16
Google scholar (150 hit)	(“health system” OR “healthcare system” OR “service delivery” OR “health information systems” OR “access to essential medicines” O “health workforce” OR “leadership and governance” OR “healthcare financing”) AND (“crisis” OR “emergency” OR “disaster” OR “armed conflict” OR “violence” OR “humanitarian crisis” OR “complex emergency”) AND (“Ethiopia” OR “Federal Democratic Republic of Ethiopia” OR “Ethiopian”)	197

### Study selection process

**Fig. 1:**
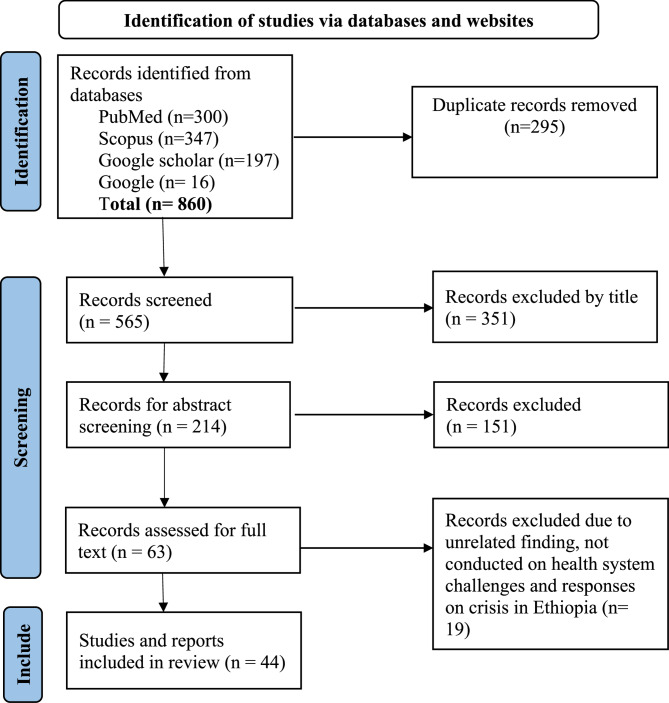
PRISMA article selection process adapted from PRISMA 2020 for new systematic reviews which included searches of databases

Three authors (AA, ED and GT) conducted the initial search in the identified databases and exported it to the EndNote reference manager to remove duplicates. Then, two independent authors (ED and GT) screened potentially eligible studies on the basis of their title and abstract to obtain potentially eligible full-text papers for further evaluation. Finally, the full texts of the potential studies selected were reviewed for inclusion by two authors (AA and YSP) on the basis standardized screening criteria. All eligibility issues raised for the study were resolved by consensus between the two investigators (AA and YSP). The PRISMA-ScR principles have been followed for the presentation of the number of items included and excluded in a flow chart (Fig. 1) [26].

### Data extraction and framework for synthesis

GT and ED extracted data from all eligible studies using a developed template. Any discrepancies between the two authors have been resolved through discussion or consultation with AA. The senior authors (AA and YSP) verified the information extracted. The main categories of extracted data were the author/s, the purpose of the study, the year of publication, the study design, the sample size (number of participants), and key summary findings. Accordingly, among the included 44 articles, reports and records, 28 were crosse-sectional studies, 8 reviews (scoping and systematic), 1 was case study and 6 were unspecified designs. In the same way, methodologically; 19 articles were quantitative, 12 were qualitative, 7 were mixed and the remaining 6 were unspecified by method (supplementary file 2).

To synthesize the findings, we applied deductive approach using the World Health Organization’s (WHO) health system building blocks framework, including service delivery, health workforce, health information systems, medicines and infrastructures, healthcare financing, and leadership and governance as a guide [27]. The extracted data were synthesized using a descriptive analytical method, and a narrative synthesis to summarize and interpret the findings. The analysis focused on: identifying common gaps across the six building blocks, understanding the relationships between different building blocks during crisis, and assessing the implications of identified gaps on health outcomes and the resilience of the system.

## Results

We retrieved a total of 860 records from the initial literature search. After removing 295 duplicates, 565 documents remained for title screening. Of these, 351 were excluded, leaving 214 for abstract review. Following abstract screening, 151 were excluded and 63 were retained for full-text review. Ultimately, 44 articles met the inclusion criteria and were included in the scoping review. The screening and selection process is shown in the PRISMA flow diagram (Fig. 1).

Our findings are presented according to the WHO health system building block frameworks. The distribution of articles across themes was as follows: eleven articles under the health service delivery theme, seven articles under the medical products, vaccines and technology theme, one articles under the health information systems (HIS) theme, fifteen articles under the human resources for health theme, five articles under the health leadership and governance theme, and five articles under the health financing theme.

### Health service delivery during crisis

A total of eleven articles were included in the health service delivery theme. Among these, one article reported that internal conflicts, the COVID-19 pandemic, and natural disasters occurred in Ethiopia over the past five years, disrupting the health system and negatively impacting the accessibility and quality of healthcare services [28].

Three reviewed articles found that the COVID-19 pandemic disrupted routine health service delivery. Inpatient, outpatient, emergency, elective surgery, and maternal-child healthcare services declined due to the crisis [29–31]. Another three articles also identified key challenges: decreased demand for healthcare services, diversion of medical supplies and health workers to COVID-19 treatment sites, movement and transportation restrictions, and insufficient health system resilience [32–34]. Similarly, reports showed that shortages of emergency medical supplies, ventilators, equipment, oxygen, and well-ventilated isolation rooms as critical barriers to service delivery during the pandemic [33, 35].

The internal conflict between Tigray region and Ethiopia’s federal government also severely impacted the health system. One study found that over three-fourths of health facilities in Tigray were partially or completely destroyed [36]. This destruction extended to Amhara and Afar regions, where facilities were damaged, destroyed, or forced to close [37]. Another article highlighted additional challenges: health worker evacuations, medical supply shortages, and transportation instability during the conflict [17].

### Medical products, vaccines and technologies during crisis

Ethiopia continues to face challenges in maintaining sustainable access to essential medicines, vaccines, and health technologies. Despite recent advancements, the convergence of the COVID-19 pandemic, internal conflicts, and natural disasters has revealed critical system vulnerabilities requiring urgent intervention [33]. Seven articles addressed medical products, vaccines, and technologies. Studies showed that COVID-19 pandemic exacerbated shortages of essential chronic medicines, resulting in higher stock-out rates and price increases for some products, shortage of personal protective equipment (PPE) and dissatisfaction of healthcare providers with availability of PPE [38, 39]. Three articles identified transportation and logistics disruptions, shortages of raw materials and active pharmaceutical ingredients, border closures and export restrictions, increased demand for specific medications, and workforce shortages caused by illness or quarantine as the major challenges of the health systems [38, 40, 41].

The armed conflict in northern Ethiopia also severely disrupted medicine, equipment, and technology supplies. One study reported that shortages of medical supplies forced the reuse of single-use items like gloves, surgical materials, and chest drains [14]. Another study revealed that in conflict-affected regions (Amhara, Tigray, and Afar), patients receiving expired medications, oxygen plants were non-functional, and routine vaccination services were suspended at some facilities [42]. Similarly, articles also highlighted political instability, disruptions of supply chains, trained staff shortages, currency inflation, and limited drug financing as barriers to obtaining essential medical supplies and vaccines during the conflict [38]. One of the reviewed articles also showed that COVID-19 disrupted BCG (62.1%), OPV (48%), IPV (40.4%), and PENTA (36.9%) vaccine availabilities [43].

### Health information systems during crisis

Crises have substantial direct and indirect effects on national health information systems (HIS), including disruptions to health service monitoring, coverage evaluation, and disease surveillance. Limited studies address HIS functionality during crises. A JSI report identified key challenges during the Northern Ethiopia conflict including destruction of HIS infrastructure, damage or loss of patient records, financial documents, logistics files, and administrative archives. These losses subsequently impede disease and service trend monitoring, epidemic identification and outbreak prediction, health commodity forecasting, quality health service provision. Furthermore, HIS infrastructure was destroyed either intentionally or unintentionally by combatants. Consequently, reporting and program monitoring activities were restricted to safe, and accessible area [34]. The use of paper-based pharmaceutical services, patient records, and reports was a drawback of the Ethiopian health system, and it was doubly challenged during the crisis. A mixed study identified limiting effective coordination like limited functionality of digital health systems, incompatible data formats, and financial constraints and socio-cultural barriers as constraints of information sharing across institutions in Ethiopia during public health emergencies [44].

### Health workforce

Fifteen documents highlighted significant human resource for health (HRH) gaps in Ethiopia’s health system during natural disasters and armed conflicts. Accordingly, the most commonly identified gaps in the health workforce included a shortage of adequately trained healthcare professionals [45–47], insufficient ongoing professional development [45, 48], and high turnover rates [45, 49, 50].

For instance, the Tigray conflict highlighted severe shortages in medical staff, which hampered the ability to provide essential health services [51]. This crisis was compounded by health worker emigration and inadequate domestic training capacity. HRH scarcity compromised essential care delivery, contributing to increased population morbidity and mortality.

Another critical gap was the lack of continuous professional development and crisis-specific training [52]. This deficiency impaired healthcare workers’ ability to adapt to emergency protocols [53], compromising care quality and increasing infection risks [54].

Furthermore, high turnover rates driven by low salaries, poor working conditions, and limited career advancement were consistently documented [49, 55–57]. These factors demoralized the workforce and diminished crisis response capacity. Current conflicts in Amhara and Oromia regions have further degraded working conditions, exacerbating turnover and absenteeism [58, 59].

### Leadership and governance

Regarding leadership and governance, five studies described healthcare leadership and governance related gaps during crisis in Ethiopia. Countries need agile, adaptable, and transformational, collaborative, multi-level, smart and ethical governance to effectively respond to emerging and re-emerging public health threats. However, the country’s governance frameworks and measures are inadequate for understanding national governance of the COVID-19 epidemic [60]. A significant gap identified in our review was the weak coordination and communication between various levels of government and health institutions [61, 62]. This poor coordination leads to delayed responses and inefficient resource allocation during crises. For example, during the COVID-19 pandemic, the slow dissemination of information and unclear communication channels hindered timely decision-making and implementation of response strategies [18]. Additionally, there were discrepancies between health policies and their actual implementation, often due to administrative bottlenecks and a lack of accountability. Many health institutions lacked comprehensive emergency preparedness plans, leading to disorganized responses and inadequate resource management during emergencies [63]. For example, poor enforcement of infection control policies contributed to the spread of diseases within healthcare settings during the COVID-19 crisis [54, 64].

### Health financing

In the area of health financing, five records reported about the gaps of health financing issues during the crisis in the Ethiopia. Accordingly, one of the most significant gaps identified is insufficient financial resources allocation for health services [65, 66]. This underfunding has been particularly evident during the COVID-19 pandemic, where the lack of sufficient financial resources impeded the procurement of essential medical supplies maintenance of facilities, and support for healthcare workers [66].

Another critical gap that hindered effective crisis response is the inefficient use of available financial resources including mismanagement and lack of transparency in the allocation and utilization of funds [18, 67]. This inefficiency often results in resources not reaching the areas of greatest need, thereby undermining the effectiveness of crisis response efforts. For instance, during the COVID-19 crisis, delays and misallocation of funds led to shortages of personal protective equipment (PPE) and other essential supplies [18, 68].

Additionally, dependency of the health system on external aid and donor funding is a critical challenge, which makes it vulnerable during crises when these funds may not be readily available or may come with restrictive conditions [69]. This dependency limits the ability of the health system to respond promptly and effectively to emergencies. For example, during the COVID-19 pandemic, the delays in receiving external funds and the conditions attached to them significantly hampered the response efforts supplies [18].

## Discussion

Ensuring the availability and delivering healthcare services with maximum quality standards is one of the key functions of the healthcare system in any country. This scoping review analyzed 44 articles on Ethiopia’s health system gaps and response during crises (COVID-19, conflict, and other disasters) using the WHO health system building blocks framework. Key findings revealed severe disruptions in service delivery, including reduced access to routine care due to resource shifts and facility damage, particularly in conflict-affected regions like Tigray, Amhara and Afar. Stockouts of medical supplies, expired medication use, supply chain breakdowns, destruction of HIS infrastructure and paper-based record-keeping, shortages of health work force, inadequate training, and high turnover exacerbated by poor working conditions, weak coordination, slow crisis responses, poor policy implementation, underfunding, inefficient resource use, and overreliance on unstable external aid were the identified gaps during crisis in the Ethiopian health system. Collectively, these challenges underscored systemic vulnerabilities, highlighting the need for resilient infrastructure, better workforce support, and sustainable financing to mitigate future crises.

Most of the articles reviewed under health service delivery theme of this study were found that both COVID-19 pandemic and the internal conflict happened in the northern Ethiopia compromised outpatient, inpatient, surgical, maternal and child health service quality and accessibility. The findings were supported by the study done in economically developed multi-countries involved study that found COVID-19 outbreak was associated with a significant reduction in hospital admissions [70]. Similar studies conducted in war affected countries, Afghanistan, and South Sudan, also supported our findings. The studies found that reproductive, maternal and child health service delivery has been significantly hampered by conflict [71, 72]. This implied that crises deteriorated the quality and extent of healthcare service deliveries [73]. Our study also revealed that the health service delivery at a time of crisis was challenged by evacuation of health personnel, shortage of medical supplies, lack of well-designed health facilities and transportation which is in line with previous studies. Conflict damages health and health-related infrastructure and leads to shortages in medicines, medical supplies, health personnel, and financial resources. The cholera pandemic in the Yemen showed that the crisis compromised reproductive, maternal and child health service [74, 75].

Our study highlighted those essential chronic medicines had higher rates of stock-outs and increased unit price during COVID-19 crisis. It was supported by the study done in Aden which stated that during crisis the price of life saving medicines increased by over 71% [76].|Our study also showed that the Ethiopian health system was also challenged by disruptions in transportation and logistics, shortages of raw materials and active pharmaceutical ingredients, border closures and export restrictions, increased demand for certain medications and workforce shortages due to illness or quarantine measures which is supported by studies done in India and South Sudan. Shortage of drugs, medicines, and medical supplies following the conflict in south Sudan and India due to worsen economic condition, inappropriate pricing policy, privatization of the pharmaceutical sector, poor manufacturing and weak regulatory system [77, 78].

Our study also found that in the war affected regions of Ethiopia, gloves, surgical materials and chest drains were being washed and re-used, oxygen plants were not working, and some health facilities couldn’t provide routine vaccines and use expired medications. It was also highlighted that political instability, shortage of trained human resources, currency inflation, and limited drug financing were the challenges to avail the required medical supplies, equipment and technologies during crisis which was in line with studies done in Syria and South Sudan [79, 80].

Health information system is one of the WHO health systems building blocks disrupted at a time of crisis. Since most of the health facilities in Ethiopia uses paper based pharmaceutical services, patient records, and reports, and the HIS was destructed and easily collapsed by the war and back-up records were not found at post-war phase. The Ethiopian northern war destructed of HIS infrastructures, damaged or misplaced of patient, financial, logistic, and administrative records. The finding was congruent with a study done in conflict zones that found obstruction of healthcare delivery, incursions into, looting of, and damage to health facilities were some of the challenges of HIS in conflict affected zones [81].

In health financing, one of the most critical gaps identified in this review was the insufficient allocation of financial resources for health services. This underfunding was particularly evident during the COVID-19 pandemic, where the lack of adequate financial resources severely impacted the procurement of essential medical supplies, the maintenance of healthcare facilities, and the support provided to healthcare workers. This finding was supported by existing literature on health financing in low- and middle-income countries (LMICs) during crises [82, 83]. This under funding in health systems often leads to inadequate crisis preparedness and response.

Inefficient use of available financial resources including issues such as mismanagement and a lack of transparency in fund allocation and utilization was another major gap identified in this review. This finding was consistent with similar studies in Pakistan and Malawi which documented the challenges of financial mismanagement and lack of transparency in the health sector, particularly during crisis situations when rapid and effective resource allocation is critical [84, 85]. These issues are often compounded by weak governance structures that fail to ensure accountability in the use of funds [84].

Additionally, this review identified the Ethiopian health system’s heavy dependency on external aid and donor funding as a critical challenge. This reliance made the system particularly vulnerable during crises when external funds may be delayed or come with restrictive conditions. The finding was supported by findings from previous studies on health financing in LMICs [86]. This implied the need for more sustainable and autonomous financing mechanisms within the health system is crucial.

This study also highlighted weak coordination and communication between various levels of government and health institutions being a predominant challenge of the Ethiopian health system during crisis. This finding was consistent with other similar studies that identified weak communication networks and poor inter-agency coordination during the early stages of the COVID-19 pandemic significantly contributed to the global spread of the virus [87, 88]. Similarly, the World Health Organization (WHO) has long advocated for clear communication strategies and robust coordination mechanisms as essential components of effective emergency preparedness and response [89].

Our review also indicated discrepancies between health policies and their actual implementation. This finding aligns with similar study that highlighted the challenges of translating health policies into actionable plans, particularly in resource-constrained settings where administrative inefficiencies and lack of accountability are prevalent [90].

The review also revealed that many health institutions lacked comprehensive emergency preparedness plans, contributing to disorganized responses and poor resource management. For instance, inadequate enforcement of infection control policies within healthcare settings during the COVID-19 crisis exacerbated the spread of the virus. Similar report on the Ebola outbreak highlighted that the absence of robust preparedness plans significantly weakened the effectiveness of the health system’s response [91]. This implied the critical role of preparedness and the consequences of its absence during crises.

This review identified a significant shortage of healthcare professionals, exacerbated by the migration of health workers to other countries and inadequate domestic training capacities. Studies have frequently highlighted similar issues, including shortages of trained professionals and inadequate training during emergencies [92, 93]. The high turnover rates due to poor working conditions and low salaries are also well-documented in the literature, reflecting a global trend observed in various low-resource settings [94]. Lack of continuous professional development and crisis-specific training for healthcare workers emerged as a critical issue. This gap hampered healthcare workers’ ability to adapt to new protocols and treatment guidelines during emergencies, compromising the quality of care and increasing the risk of healthcare-associated infections [94].

### Policy and research implications

This scoping review addresses critical evidence gaps in understanding how Ethiopia’s health system performs during crises (such as COVID-19, and conflict) by synthesizing data from 43 studies using the WHO health system building blocks framework. Unlike prior fragmented studies, this study was analyzed holistically how multiple crises disrupt all health system components. The findings of our research will be used for policy makers, project designers, researchers and other humanitarian organizations to prioritize context-specific solutions and assess multi-crisis health system resilience in Ethiopia.

### Forwarded recommendations

The government and minister of health need to establish flexible and scalable emergency plans for essential health services during emergencies. Strengthen the primary health healthcare and community health services deliveries may help to address problem of accessing basic health services during crisis. Development of interoperable HIS, which could integrate data from multiple sources and be used by multiple organizations, and equipping the facilities with trained professionals are also our recommendations. Increasing investment in training and retention of health workers, improving working conditions, and providing targeted crisis-specific training programs are critical steps that will help improve response to crises. It is also important to reducing dependency on external aid and increase domestic funding for health, and ensuring efficient and transparent use of healthcare resources to improve the preparedness and response of the health system to any future crisis.

### Strength and limitations

The study entails evidences and challenges of the health system with its building blocks from both man-made and natural crisis perspectives, which yields more comprehensive evidences. However, the review is prone to publication bias, whereby studies with significant or positive results are more likely to be published; it may have overlooked other relevant challenges or gaps. This study also used only two databases and grey literature and includes only studies from 2020 to 2024 conducted in Ethiopia. Since health crises are dynamic, some of the challenges or evidence gaps identified may change over time, and the updates should be continuous to remain relevant.

## Conclusions

As evidenced from our review, the health system was challenged both during man-made and natural crisis. The COVID-19 pandemic and the northern Ethiopia internal conflict disturbed the routine health service delivery, destructed the health infrastructures and the health information system and lead to supply run out. Shortage of emergency medical supplies, medical equipment, lack of transportation, medical cost inflation and poor infrastructure health facilities were some challenges to deliver healthcare services during crisis.

## Supplementary Information

Supplementary Material 1.

Supplementary Material 2.

## Data Availability

Data is available online and unpublished reports can be accessed based on reasonable requests from primary and correspondent authors.

## References

[1] Brecher M, Wilkenfeld J. A study of crisis. University of Michigan Press; 2022.

[2] Bookchin M. The modern crisis. AK Press; 2022.

[3] Kentikelenis A, Papanicolas I. Economic crisis, austerity and the Greek public health system. Eur J Public Health. 2012;22(1):4–5. 10.1093/eurpub/ckr190 22199160

[4] Kieny M-P, Evans DB, Schmets G, Kadandale S. Health-system resilience: reflections on the Ebola crisis in western Africa. SciELO Public Health. 2014;92:850–850. 10.2471/BLT.14.149278 PMC4264399 25552765

[5] Massuda A, Hone T, Leles FAG, De Castro MC, Atun R. The Brazilian health system at crossroads: progress, crisis and resilience. BMJ Global Health. 2018;3(4):e000829. 10.1136/bmjgh-2018-000829 PMC6035510 29997906

[6] Jordan K, Lewis TP, Roberts B. Quality in crisis: a systematic review of the quality of health systems in humanitarian settings. Confl Health. 2021;15:1–13. 33531065 10.1186/s13031-021-00342-z PMC7851932

[7] Martineau T, McPake B, Theobald S, Raven J, Ensor T, Fustukian S, Ssengooba F, Chirwa Y, Vong S, Wurie H. Leaving no one behind: lessons on rebuilding health systems in conflict-and crisis-affected States. BMJ Global Health. 2017;2(2):e000327. 10.1136/bmjgh-2017-000327 PMC5656126 29082000

[8] Brunn M, Brigham KB, Chevreul K, Hernández-Quevedo C. The impact of the crisis on the health system and health in France. Economic Crisis, Health Systems and Health in Europe. European Observatory on Health Systems and Policies; 2015.

[9] Lindelow M, Serneels P. The performance of health workers in Ethiopia: results from qualitative research. Soc Sci Med. 2006;62(9):2225–35. 16309805 10.1016/j.socscimed.2005.10.015

[10] Erena SH, Worku H. Urban flood vulnerability assessments: the case of dire Dawa city, Ethiopia. Nat Hazards. 2019;97:495–516.

[11] Gebrehiwot T, van der Veen A. Climate change vulnerability in Ethiopia: disaggregation of Tigray region. J East Afr Stud. 2013;7(4):607–29.

[12] Ahmad T, Cressman K, Noorka IR, Omrane MB, Bader MK. Burgeoning desert locust population as a transboundary plant pest: A significant threat to regional food security. The Food Security, Biodiversity, and Climate Nexus. Springer. 2022;pp.189–212.

[13] Berhan Y, Assefa B, Tassew A, Mengiste W, Gebreyesus A, Geletu Z, Muluneh K, Asfaw G, Abera S, Negesso A. The protracted civil and armed conflicts in Ethiopia fueling the COVID-19-related health crisis: perspective on Building a resilient health system to shocks. Ethiop J Health Sci. 2023;33(5):869–80. 38784511 10.4314/ejhs.v33i5.17 PMC11111203

[14] Arage MW, Kumsa H, Asfaw MS, Kassaw AT, Dagnew EM, Tunta A, Kassahun W, Addisu A, Yigzaw M, Hailu T. Exploring the health consequences of armed conflict: the perspective of Northeast Ethiopia, 2022: a qualitative study. BMC Public Health. 2023;23(1):2078. 37875885 10.1186/s12889-023-16983-z PMC10594710

[15] Gutema G, Kaba M, Birhanu Z, Diribi J, Elemo I. Impact of armed conflicts on public health infrastructure and services in oromia, Ethiopia. Cureus. 2023;15(6):e40653. 10.7759/cureus.40653 PMC10356178 37476107

[16] Tekulu FB, Gebre HT: Effect of War on Health Institutions of Eastern Tigray, Ethiopia. Public Health Challenges. 2025;4:e70063.

[17] Arage MW, Kumsa H, Asfaw MS, Kassaw AT, Mebratu E, Tunta A, Kassahun W, Adissu A, Yigzaw M, Hailu T,Tenaw LA. Assessing the health consequences of northern Ethiopian armed conflict. J Public Health Policy. 2024;45(1):43–57. 10.1057/s41271-023-00464-z PMC10920422 38310169

[18] Abagero A, Ragazzoni L, Hubloue I, Barone-Adesi F, Lamine H, Addissie A, Della Corte F, Valente M. A review of COVID-19 response challenges in Ethiopia. Int J Environ Res Public Health. 2022;19(17):11070. 36078785 10.3390/ijerph191711070 PMC9518440

[19] Taye A, Haile Mariam D, Murray V. Interim report: review of evidence of the health impact of famine in Ethiopia. Perspect Public Health. 2010;130(5):222–6. 10.1177/1757913910379197 21086818

[20] Gudina EK, Siebeck M, Eshete MT. Evidence gaps and challenges in the fight against COVID-19 in Africa: scoping review of the Ethiopian experience. Risk Manag Healthc Policy. 2021;14:4511–21. 10.2147/RMHP.S333545 PMC8575488 34764709

[21] Barbisch D, Haik J, Tessone A, Hanfling D. Surge capacity. Koenig and Schultz’s disaster medicine: comprehensive principles and practices. 2010. p. 133.

[22] Antos JR. Health care financing in Thailand: Modeling and sustainability. In: Bangkok: Workshop on Model Development of Sustainable Health Care Financing: 2007; 2007.

[23] Hafner T, Shiffman J. The emergence of global attention to health systems strengthening. Health Policy Plann. 2013;28(1):41–50. 10.1093/heapol/czs023 22407017

[24] Arksey H, O’Malley L. Scoping studies: towards a methodological framework. Int J Soc Res Methodol. 2005;8(1):19–32.

[25] Thielen F, Van Mastrigt G, Burgers L, Bramer W, Majoie H, Evers S, Kleijnen J. How to prepare a systematic review of economic evaluations for clinical practice guidelines: database selection and search strategy development (part 2/3). Expert Rev PharmacoEcon Outcomes Res. 2016;16(6):705–21. 27805466 10.1080/14737167.2016.1246962

[26] Tricco AC, Lillie E, Zarin W, O’Brien KK, Colquhoun H, Levac D, Moher D, Peters MD, Horsley T, Weeks L. PRISMA extension for scoping reviews (PRISMA-ScR): checklist and explanation. Ann Intern Med. 2018;169(7):467–73. 30178033 10.7326/M18-0850

[27] World Health Organization. Monitoring the Building blocks of health systems: a handbook of indicators and their measurement strategies. World Health Organization; 2010.

[28] Abraha HE, Tequare MH, Teka H, Gebremedhin MB, Desta KG, Ebrahim MM, Yemane A, Gebremariam SM, Gebresilassie KB, Tekle TH. Impact of a double catastrophe, war and COVID-19, on health service utilization of a tertiary care hospital in tigray: an interrupted time-series study. Confl Health. 2023;17(1):37. 37580780 10.1186/s13031-023-00537-6 PMC10426210

[29] Shuka Z, Mebratie A, Alemu G, Rieger M, Bedi AS. Use of healthcare services during the COVID-19 pandemic in urban Ethiopia: evidence from retrospective health facility survey data. BMJ Open*. *2022;12(2):e056745. 10.1136/bmjopen-2021-056745 PMC8882656 35197352

[30] Shimels T. The Trend of Health Service Utilization and Challenges Faced During the COVID-19 Pandemic at Primary Units in Addis Ababa: A Mixed-Methods Study. Health Serv Res Manag Epidemiol*. *2021;8:23333928211031119. 10.1177/23333928211031119 PMC8273870 34291123

[31] Tefera YG, Ayele AA. Newborns and Under-5 Mortality in Ethiopia: The Necessity to Revitalize Partnership in Post-COVID-19 Era to Meet the SDG Targets. J Prim Care Community Health. 2021;12:2150132721996889. 10.1177/2150132721996889 PMC7917850 33632030

[32] Birihane BM, Bayih WA, Alemu AY, Belay DM. Perceived Barriers and Preventive Measures of COVID-19 Among Healthcare Providers in Debretabor, North Central Ethiopia, 2020. Risk Manag Healthc Policy. 2020;13:2699–2706. 10.2147/RMHP.S287772 PMC7685372 33244283

[33] Abagero A, Ragazzoni L, Hubloue I, et al. A Review of COVID-19 Response Challenges in Ethiopia. Int J Environ Res Public Health. 2022;19(17):11070. 10.3390/ijerph191711070 PMC9518440 36078785

[34] Taye M, Medhanyie AA, Van Reisen M. War-related destruction of the digital health data infrastructure: discovering features for a resilient digital health information system. Tigray War Digit Black Hole. 2024:439–476.

[35] Gesesew H, Berhane K, Siraj ES, Siraj D, Gebregziabher M, Gebre YG, Gebreslassie SA, Amdeslassie F, Tesema AG, Siraj A. The impact of war on the health system of the Tigray region in Ethiopia: an assessment. BMJ Global Health. 2021;6(11):e007328. 10.1136/bmjgh-2021-007328 PMC8611430 34815244

[36] Gufue ZH, Haftu HK, Alemayehu Y, Tsegay EW, Mengesha MB, Dessalegn B. Damage to the public health system caused by war-related looting or vandalism in the Tigray region of Northern Ethiopia. Front Public Health. 2024;12:1271028. 10.3389/fpubh.2024.1271028 PMC11026641 38645448

[37] Damtew AW, Ejigu AA. Study the facts and truth on the levels of conflict disasters and victims: in the case of Amhara and Afar-Ethiopia. Ethiopian Journal of Social Sciences. 2022;8(2):68–96.

[38] Mekonnen Z, Melaku T, Tucho GT, Mecha M, Årdal C, Jahre M. The knock-on effects of COVID-19 pandemic on the supply and availability of generic medicines in Ethiopia: mixed methods study. BMC Health Serv Res. 2023;23(1):513. 10.1186/s12913-023-09535-z PMC10199739 37210502

[39] Deressa W, Worku A, Abebe W, Gizaw M, Amogne W. Availability and use of personal protective equipment and satisfaction of healthcare professionals during COVID-19 pandemic in addis ababa, Ethiopia. Archives Public Health. 2021;79:1–14. 10.1186/s13690-021-00668-3 PMC8369137 34404464

[40] Melaku T, Mekonnen Z, Terefe Tucho G, Mecha M, Årdal C, Jahre M. Availability of essential, generic medicines before and during COVID-19 at selected public pharmaceutical supply agencies in Ethiopia: a comparative cross-sectional study. BMJ Open. 2024;14(3):e077545. 10.1136/bmjopen-2023-077545 PMC11146403 38443082

[41] Ejeta F, Feyisa D, Kebede O, Mechessa DF, Zewudie A, Mamo Y, Regasa T, Abebe L. Logistics management of Covid-19 personal protective equipment and its challenges at public hospitals of Southwest Ethiopia: an integrated quantitative and qualitative study. International Journal of Medical Research & Health Sciences. 2021;10(7):178–185

[42] cross IcoR. Dwindling medical supplies in northern Ethiopia prevents health workers from aiding those in need. Accessed 08 Aug 2024. Available at https://www.icrc.org. 2022.

[43] Adilo TM, Endale SZ, Demie TG, Dinka TG. The Impact of COVID-19 on Supplies of Routine Childhood Immunization in Oromia Regional State, Ethiopia: A Mixed Method Study. Risk Manag Healthc Policy. 2022;15:2343–2355. 10.2147/RMHP.S386717 PMC9759002 36536936

[44] Sasie SD, Van Zuylen P, Ayano G, Aragaw FM, Spigt M. Information sharing across institutions: practices and barriers during public health emergencies in Ethiopia. Int J Med Informatics. 2024;186:105439. 10.1016/j.ijmedinf.2024.105439 38564958

[45] Firew T, Gebreyesus A, Woldeyohannes L, Ebrahim F, Patel S. Human resources for emergency care systems in Ethiopia: challenges and triumphs. Afr J Emerg Med. 2020;10:S50–5. 33318902 10.1016/j.afjem.2020.09.006 PMC7723913

[46] Zewudie A, Regasa T, Kebede O, Abebe L, Feyissa D, Ejata F, Feyisa D, Mamo Y. Healthcare Professionals' Willingness and Preparedness to Work During COVID-19 in Selected Hospitals of Southwest Ethiopia. Risk Manag Healthc Policy. 2021;14:391–404. 10.2147/RMHP.S289343 PMC7868776 33568957

[47] Mitike G, Nigatu F, Wolka E, Defar A, Tessema M, Nigussie T. Health system response to COVID-19 among primary health care units in Ethiopia: A qualitative study. PLoS ONE. 2023;18(2):e0281628. 36763695 10.1371/journal.pone.0281628 PMC9916627

[48] Khatri RB, Endalamaw A, Erku D, Wolka E, Nigatu F, Zewdie A, Assefa Y. Preparedness, impacts, and responses of public health emergencies towards health security: qualitative synthesis of evidence. Archives Public Health. 2023;81(1):208. 10.1186/s13690-023-01223-y PMC10687930 38037151

[49] Mihretie TM, Abebe GK, Mulugeta H, Kassaw AT, Alamaw AW, Adugna B, Ergetie FS, Zemariam AB. Turnover intention and associated factors among nurses working at governmental hospitals in Bahir Dar City at the time of war, Northwest Ethiopia, 2022. Int J Afr Nurs Sci. 2024;20:100724.

[50] Gebrekidan AY, Enaro EY, Azeze G, Adella GA, Kassie GA, Haile KE, Asgedom YS. Turnover intention among healthcare workers in Ethiopia: a systematic review and meta-analysis. BMJ Open. 2023;13(5):e067266. 10.1136/bmjopen-2022-067266 PMC10230902 37221024

[51] Gesesew H, Berhane K, Siraj ES, et al. The impact of war on the health system of the Tigray region in Ethiopia: an assessment. BMJ Glob Health. 2021;6(11):e007328. 10.1136/bmjgh-2021-007328 PMC8611430 34815244

[52] Nicolai S, Diwakar V, Khan A, Mansour-Ille D, Anderson A. Strengthening coordinated education planning and response in crisis contexts: Synthesis Report. Overseas Development Institute; 2020.

[53] Etafa W, Gadisa G, Jabessa S, Takele T. Healthcare workers' compliance and its potential determinants to prevent COVID-19 in public hospitals in Western Ethiopia. BMC Infect Dis. 2021;21(1):454. 34011263 10.1186/s12879-021-06149-w PMC8132019

[54] Daba C, Atamo A, Weldehanna DG, Oli A, Debela SA, Luke AO, Gebrehiwot M. Infection prevention and control compliance of healthcare workers towards COVID-19 in conflict-affected public hospitals of Ethiopia. BMJ Open. 2023;13(12):e074492. 10.1136/bmjopen-2023-074492 PMC10759124 38159945

[55] Gebregziabher D, Berhanie E, Berihu H, Belstie A, Teklay G. The relationship between job satisfaction and turnover intention among nurses in axum comprehensive and specialized hospital tigray, Ethiopia. BMC Nurs. 2020;19:1–8. 32831645 10.1186/s12912-020-00468-0 PMC7437041

[56] SENDEKIE TY, Temam G, vas Roosmalen J, Stekelenburg J, Kim YM, Shawula S, Woldemariam D, Yilma E. Satisfaction and turnover intention of physicians and public health officers in government health facilities: a National cross-sectional study. Ethiop Med J. 2020;58(01).

[57] Kassahun CW, Abate AT, Tezera ZB, Beshah DT, Agegnehu CD, Getnet MA, Abate HK, Yazew BG, Alemu MT. Working environment of nurses in public referral hospitals of West amhara, Ethiopia, 2021. BMC Nurs. 2022;21(1):167. 35751081 10.1186/s12912-022-00944-9 PMC9229886

[58] Ethiopia. Army Attacks Health Care in Amhara Conflict: https://www.hrw.org/news/2024/07/03/ethiopia-army-attacks-health-care-amhara-conflict.

[59] Ethiopia. Healthcare crisis in Oromia exacerbated by massive displacement. https://www.icrc.org/en/document/ethiopia-healthcare-crisis-oromia-exacerbated-massive-displacement.

[60] Assefa Y, Woldeyohannes S, Cullerton K, Gilks CF, Reid S, Van Damme W. Attributes of National governance for an effective response to public health emergencies: lessons from the response to the COVID-19 pandemic. J Global Health. 2022;12:05021. 10.7189/jogh.12.05021 PMC9258903 35787525

[61] Rawat A, Karlstrom J, Ameha A, et al. The contribution of community health systems to resilience: Case study of the response to the drought in Ethiopia. J Glob Health. 2022;12:14001. 10.7189/jogh.12.14001 PMC9588157 36273279

[62] Debela BK. The COVID-19 pandemic and the Ethiopian public administration: responses and challenges. Good public governance in a global pandemic. International Institute of Administrative Sciences. 2020;113–124.

[63] Zikargae MH. COVID-19 in Ethiopia: assessment of how the Ethiopian government has executed administrative actions and managed risk communications and community engagement. Risk Manag Healthc Policy. 2020;13:2803–10. 33299368 10.2147/RMHP.S278234 PMC7721303

[64] Nigussie H. The coronavirus intervention in Ethiopia and the challenges for implementation. Front Communication. 2021;6:562512.

[65] Debie A, Khatri RB, Assefa Y. Contributions and challenges of healthcare financing towards universal health coverage in Ethiopia: a narrative evidence synthesis. BMC Health Serv Res. 2022;22(1):866. 35790986 10.1186/s12913-022-08151-7 PMC9254595

[66] Ethiopian Federal Ministry of Health, Annual Health Sector Performance Report, 2021/22, Federal Ministry of Health, Ethiopia.

[67] Ethiopia Priority Humanitarian Response and Critical Funding Gaps. 2024. https://reliefweb.int/report/ethiopia/ethiopia-priority-humanitarian-response-and-critical-funding-gaps-february-2024.

[68] Wondimu W, Girma B. Challenges and Silver Linings of COVID-19 in Ethiopia -Short Review. J Multidiscip Healthc. 2020;13:917–922. 10.2147/JMDH.S269359 PMC7502404 32982268

[69] Mumin AA, Oladeji O, Yohannes A. Factors associated with utilization of donor funds in Somali region of Ethiopia. J Econ Manage Trade. 2021;27(3):65–76.

[70] Pereira H, Naber C, Wallace S, Gabor T, Abdi S, Alekyan B, Alexander T, Artucio C, Batista I, Candiello A. Stent-Save a Life international survey on the practice of primary percutaneous coronary intervention during the COVID-19 pandemic. Rev Port Cardiol. 2022;41(3):221–227. 10.1016/j.repc.2021.04.006 PMC8709833 34975228

[71] Mirzazada S, Padhani ZA, Jabeen S, et al. Impact of conflict on maternal and child health service delivery: a country case study of Afghanistan. Confl Health. 2020;14:38. 10.1186/s13031-020-00285-x PMC7288441 32536966

[72] Sami S, Mayai A, Sheehy G, Lightman N, Boerma T, Wild H, Tappis H, Ochan W, Wanyama J, Spiegel PJC, et al. Maternal and child health service delivery in conflict-affected settings: a case study example from upper nile and unity States. South Sudan. 2020;14:1–12. 10.1186/s13031-020-00272-2 PMC7254670 32514299

[73] Biel M, Grondys K, Androniceanu AM. A Crisis in the Health System and Quality of Healthcare in Economically Developed Countries. Int J Environ Res Public Health. 2022;20(1):469. 10.3390/ijerph20010469 PMC9819705 36612791

[74] Tappis H, Elaraby S, Elnakib S, et al. Reproductive, maternal, newborn and child health service delivery during conflict in Yemen: a case study. Confl Health. 2020;14:30. 10.1186/s13031-020-00269-x PMC7254736 32514295

[75] Debarre A. Hard to reach: providing healthcare in armed conflict. International Peace Institute; 20.

[76] Sallami Z, Kassim Y, Selvaraj J, Parry E, Winter G. Impact of the armed conflict of 2015–2016 in Aden on health services and the availability of medicines. Health. 2017;9(4):685–96.

[77] Dapke K, Phadke R, Rocha I, dos Santos Costa A, Ahmad S, Essar M, Menon V, Bassey E, Malhotra K, Shah J. Drug supply shortage in India during COVID-19 pandemic: efforts and challenges. HPHR. 2021;31.

[78] Lucero-Prisno III DE, Elhadi YAM, Modber MAA,Musa MB, Mohammed SEE, Hassan KF, Dafallah A, Lin X, Ahmadi A, Adeyemi S. Drug shortage crisis in Sudan in times of COVID-19. Public Health Pract (Oxf). 2020;1:100060. 10.1016/j.puhip.2020.100060 PMC9461216 36101692

[79] Khogali A, Homeida A. Impact of the 2023 armed conflict on Sudan’s healthcare system. Public Health Chall. 2023;2:e134. 10.1002/puh2.134 PMC12039653 40496780

[80] Kherallah M, Alahfez T, Sahloul Z, Eddin KD, Jamil G. Health care in Syria before and during the crisis. Avicenna J Med. 2012;2(3):51–53. 10.4103/2231-0770.102275 PMC3697421 23826546

[81] Haar R, Sirkin S. Strengthening data to protect healthcare in conflict zones. 2022.

[82] Kwon S, Kim E. Sustainable health financing for COVID-19 preparedness and response in Asia and the Pacific. Asian Economic Policy Rev. 2022;17(1):140–56.

[83] De Foo C, Verma M, Tan SY, Hamer J, van der Mark N, Pholpark A, Hanvoravongchai P, Cheh PLJ, Marthias T, Mahendradhata Y, et al. Health financing policies during the COVID-19 pandemic and implications for universal health care: a case study of 15 countries. Lancet Global Health. 2023;11(12):e1964–77. 37973344 10.1016/S2214-109X(23)00448-5 PMC10664823

[84] Nayupe SF, Munharo S, Mbulaje P, Banda C, Lucero-Prisno III DE. Covid-19 and fund mismanagement in malawi: A major challenge to its effective pandemic containment. Health Sci Rep. 2022;5(3). 10.1002/hsr2.546 PMC8986973 35415273

[85] Khan MR, Nazir MA, Afzal S, Sohail J. Health financing and public financial management during the Covid-19 pandemic: evidence from Pakistan as low-income country. Int J Health Plann Manag. 2023;38(3):847–72. 10.1002/hpm.3630 36882664

[86] Strupat C, Balasubramanian P, Srigiri SR, Hornidge A-K. Health financing in times of multiple crises: analysis and recommendations. In.: IDOS Policy Brief; 2023.

[87] Ratzan SC, Sommarivac S, Rauh L. Enhancing global health communication during a crisis: lessons from the COVID-19 pandemic. 2020. 10.17061/phrp3022010 32601655

[88] Forman R, Atun R, McKee M, Mossialos E. 12 lessons learned from the management of the coronavirus pandemic. Health Policy. 2020;124(6):577–80. 32425281 10.1016/j.healthpol.2020.05.008 PMC7227502

[89] Jee Y. WHO International Health Regulations Emergency Committee for the COVID-19 outbreak. Epidemiol Health. 2020;42:e2020013. 10.4178/epih.e2020013 PMC7285442 32192278

[90] Alves F, Leal Filho W, Casaleiro P, Nagy GJ, Diaz H, Al-Amin AQ, de Andrade JBSO, Hurlbert M, Farooq H, Klavins M. Climate change policies and agendas: facing implementation challenges and guiding responses. Environ Sci Policy. 2020;104:190–8.

[91] Abayomi A, Balogun MR, Bankole M, Banke-Thomas A, Mutiu B, Olawepo J, Senjobi M, Odukoya O, Aladetuyi L, Ejekam C. From Ebola to COVID-19: emergency preparedness and response plans and actions in lagos, Nigeria. Globalization Health. 2021;17:1–10. 34243790 10.1186/s12992-021-00728-x PMC8267235

[92] Sultan MAS, Løwe Sørensen J, Carlström E, Mortelmans L, Khorram-Manesh A. Emergency healthcare providers’ perceptions of preparedness and willingness to work during disasters and public health emergencies. Healthcare. 2020;2020:442 MDPI. 10.3390/healthcare8040442 PMC7712235 33138164

[93] Razu SR, Yasmin T, Arif TB, Islam MS, Islam SMS, Gesesew HA, Ward P. Challenges faced by healthcare professionals during the COVID-19 pandemic: a qualitative inquiry from Bangladesh. Front Public Health. 2021;9:647315. 34447734 10.3389/fpubh.2021.647315 PMC8383315

[94] Poon Y-SR, Lin YP, Griffiths P, Yong KK, Seah B, Liaw SY. A global overview of healthcare workers’ turnover intention amid COVID-19 pandemic: a systematic review with future directions. Hum Resour Health. 2022;20(1):70. 36153534 10.1186/s12960-022-00764-7 PMC9509627

