# Supplementary material for: Exploring health system challenges and gaps for crisis response in Ethiopia: a scoping review of publications and reports from 2020-2024
Source: BMC Health Serv Res. 2025 Jul 4;25:928. doi: 10.1186/s12913-025-13084-y (PMC12228147; doi:10.1186/s12913-025-13084-y)
Supplement: Supplementary file 1 — Supplementary Material 1. [file 12913_2025_13084_MOESM1_ESM.docx]

**Annex – I: Summary Table of included articles**

| Health system challenges, crisis response, and evidence gaps towards crisis in Ethiopia | | | | | | |
| --- | --- | --- | --- | --- | --- | --- |
| **Author (year). Title** | **Source/ Journal** | **Aim of the study** | **Design** | **Sample size and study participants if applicable** | **Publication date/year** | **Key Findings** |
| **Health service delivery (11)** | | | | | | |
| 1. Arage, M.W., Kumsa, H., Asfaw, M.S., Kassaw, A.T., Mebratu, E., Tunta, A., Kassahun, W., Adissu, A., Yigzaw, M., Hailu, T. and Tenaw, L.A., 2024. Assessing the health consequences of northern Ethiopian armed conflict, 2022. | Journal of public health policy | To assess the health consequences of the northern Ethiopian conflict, 2022 | mixed method study with a retrospective cross-sectional design | 1806 individuals from 423 households and 100 in-depth interviews and focused group discussion | Feb 3, 2024 | - The collapse of the health system, evacuation of health personnel, and shortage of medical supplies, and instability with a lack of transportation were the identified consequences of the conflict. - The conflict has also breakdown the health system and health-supporting structures. |
| 1. Shuka Z, Mebratie A, Alemu G, Rieger M, Bedi ASJBo (2022). Use of healthcare services during the COVID-19 pandemic in urban Ethiopia: evidence from retrospective health facility survey data | BMJ open | To explore whether the onset and spread of COVID-19 shifted the health service attention at the cost of non-Covid19 related healthcare services. | Facility based cross-sectional study design | MCH service users including family planning, antenatal postnatal, abortion care, delivery and immunization and adults attending at facilities for antiretroviral therapy (ART), tuberculosis (TB) and leprosy and dental services within the 59 health centres and 29 public hospitals during the study time | Feb 1, 2022 | - Although there is a sharp reduction in the use of inpatient and outpatient services except MCH services. - The results underline the resilience and agility displayed by one of the world's most resource-constrained healthcare systems. |
| 1. Shimels, T. (2021). The trend of health service utilization and challenges faced during the COVID-19 pandemic at primary units in Addis ababa: a mixed-methods study | Health services research and managerial epidemiology journal | To assess the trend of health service utilization and challenges faced during the COVID-19 pandemic at primary units in Addis Ababa, Ethiopia. | A mixed-methods cross-sectional design | 5 Health centers and 20 key informants from disease prevention, OPD, IPT, and pharmacy units in the selected facilities | July 8, 2021 | - The COVID-19 wave has negatively impacted the inpatient flow of some units such as PICT, VCT, FP services, and most sub-units of the OPDs. - Challenges such as fear of infection and stigma, poor infrastructure, human resources, and challenges related to the supply of prevention and treatment inputs were prominently encountered at the health centers. |
| 1. Tefera, Y. G., & Ayele, A. A. (2021). Newborns and Under-5 mortality in Ethiopia: the necessity to revitalize Partnership in Post-COVID-19 era to meet the SDG targets | Journal of primary care & community health | To assess the necessity to revitalize partnership in Post-COVID-19 era to meet the SDG targets | Review and evidence synthesis | Unspecified number of Published articles and reports | February 25, 2021 | - The COVID-19 epidemic has already eroded the health systems by disrupting the routine health services - Interruption of newborn care, prevention, and treatment of childhood illnesses including immunization services were affected due to COVID 19 epidemic |
| 1. Birihane, B. M., Bayih, W. A., Alemu, A. Y., & Belay, D. M. (2020). Perceived barriers and preventive measures of COVID-19 among healthcare providers in Debretabor, North Central Ethiopia | Risk management and healthcare policy journal | To assess perceived barriers and preventive measures of COVID 19 among healthcare providers in Debretabor Town, north central Ethiopia. | An institution-based cross-sectional survey | 183 healthcare providers | Nov 20, 2020 | - The overall preventive practice was 68.3%. - Insufficient training, lack of policy, less commitment to infection control, and limitations of resources were perceived as major barriers for practicing preventive measures during the pandemic. |
| 1. Abagero, A., Ragazzoni, L., Hubloue, I., Barone-Adesi, F., Lamine, H., Addissie, A., ... & Valente, M. (2022). A review of COVID-19 response challenges in Ethiopia. | International Journal of Environmental Research and Public Health | To describe the healthcare response challenges during the pandemic in Ethiopia. | systematic review and synthesis | 17 Published articles | September 04, 2022 | - Financial constraints led to a shortage of mechanical ventilators. - The pandemic hindered the capacity to avail full packages of personal protective equipment in health facilities and intensive care capacity. - It also affected the delivery of maternal, child and new-born services, prevention, and treatment of childhood illness, including immunization services. |
| 1. Taye, Maleda, Araya Abrha Medhanyie, and Mirjam Van Reisen. "War-related destruction of the digital health data infrastructure: Discovering features for a resilient digital health information system." | War in a Digital Black Hole, Book. | To assess the cause of the destruction of  the digital health system in the Tigray war and  the requirements for rebuilding a resilient digital health  data system | explorative participatory research with a  case study design | N/A | Nov, 2024 | - The war in Tigray the health data recording and reporting systems were destroyed. - A decline in the quality and quantity of health data collected, and interruptions to digital health systems, the destruction of patient data loss of healthcare workers and services at health facilities lack of functioning of HISs in the context of the war, siege and internet and communication blockade |
| 1. Gufue, Z.H., Haftu, H.K., Alemayehu, Y., Tsegay, E.W., Mengesha, M.B. and Dessalegn, B., 2024. Damage to the public health system caused by war-related looting or vandalism in the Tigray region of Northern Ethiopia. | Frontiers in Public Health | To estimate the cost of war-related looting or vandalism in Tigray’s public health system in Northern Ethiopia in 2021 | A retrospective cross-sectional | 936 health facilities and administrative health offices | April 04, 2024 | - The direct economic loss to the health system in monetary terms was more than $511 million. - 514 (80.6%) health posts, 153 (73.6%) health centers, 16 (80%) primary hospitals, 10 (83.3%) general hospitals, and 2 (100%) specialized hospitals were damaged and/or vandalized either fully or partially due to the war |
| 1. Damtew, A.W. and Ejigu, A.A., 2022. Study the facts and truth on the levels of conflict disasters and victims: in the case of Amhara and Afar-Ethiopia | Ethiopian Journal of Social Sciences | To investigate the facts and truth about the levels of displaced, damaged, and victims of the current Ethiopian conflict in general and the Amhara and Afar Regions in particular | exploratory qualitative methods and cross-sectional design | 382 civilians | Feb 8, 2023 | - Damaged and pillaged institutions, towns, and other public organizations such as churches, mosques, market places, educational centers, health centers, and manufacturing industries are affecting in Amhara and Afar regions - The elderly, women, youths, and children were the first victims of the conflict |
| 1. Gesesew, H., Berhane, K., Siraj, E.S., Siraj, D., Gebregziabher, M., Gebre, Y.G., Gebreslassie, S.A., Amdeslassie, F., Tesema, A.G., Siraj, A. and Aregawi, M., 2021. The impact of war on the health system of the Tigray region in Ethiopia: an assessment. | BMJ global health | To examine the extent of the health system, collapse between November 2020 and June 2021 and compare with the previous year (prewar state). | Facility based evidence analysis (quantitative cross-sectional assessment) |  | Nov. 23, 2021 | - the prewar performance of antenatal care, supervised delivery, postnatal care and children vaccination was 94%, 73%, 63% and 73%, respectively, but none of the services were likely to be delivered in the first 90 days of the war. - Six months into the war, only 27.5% of hospitals, 17.5% of health centres, 11% of ambulances and none of the 712 health posts were functional. |
| 1. Abraha, H.E., Tequare, M.H., Teka, H., Gebremedhin, M.B., Desta, K.G., Ebrahim, M.M., Yemane, A., Gebremariam, S.M., Gebresilassie, K.B., Tekle, T.H. and Atsbaha, M.T., 2023. Impact of a double catastrophe, war and COVID-19, on health service utilization of a tertiary care hospital in Tigray: an interrupted time-series study. | BMC conflict and health journal | To determine the effect of armed conflict and the COVID-19 pandemic on health service utilization at Ayder Comprehensive Specialized Hospital, in the Tigray region of Ethiopia | interrupted time-series study design (quantitative study) | patients who visited the inpatient, outpatient, and emergency-room service areas | August 14, 2023 | - Immediately after COVID-19 appeared, all the service areas exhibited a significant monthly drop in visits - Controlling the effects of time and COVID-19, the war led to a significant fall in inpatient visits [-44.3%], outpatients [-52.1%], and emergency-room attendances [-45.0%] |
| **Medical products, vaccines and technologies (7)** | | | | | | |
| 1. Mekonnen, Z., Melaku, T., Tucho, G.T., Mecha, M., Årdal, C. and Jahre, M., 2023. The knock-on effects of COVID-19 pandemic on the supply and availability of generic medicines in Ethiopia: mixed methods study | BMC Health Services Research | To assess the knock-on effects of the COVID-19 pandemic on the supply availability of non-communicable chronic disease (NCD) medicines and paracetamol products in Ethiopia. | mixed methods with cross-sectional study design | 26 hospitals | May 20, 2023 | - The overall mean availability of selected basket medicines was 63.4% - The overall stock out situation in the study area has worsened during COVID-19 compared to pre-COVID-19 time. - None of the surveyed chronic disease basket medicines met the ideal availability benchmark of 80% in health facilities |
| 1. Arage, M.W., Kumsa, H., Asfaw, M.S., Kassaw, A.T., Dagnew, E.M., Tunta, A., Kassahun, W., Addisu, A., Yigzaw, M., Hailu, T. and Tenaw, L.A., 2023. Exploring the health consequences of armed conflict: the perspective of Northeast Ethiopia, 2022: a qualitative study. | BMC public health | To explore the health consequences of the northern Ethiopian conflict in the North Wollo zone, northeast Ethiopia, in 2022. | descriptive qualitative |  | October 24, 2023 | - The conflict caused a breakdown in the health system - Distraction of health infrastructure, fleeing of health workers and shortage of medication, together - Insecurity and lack of transportation, which greatly affected the provision and utilization of health services. |
| 1. Melaku, T., Mekonnen, Z., Tucho, G.T., Mecha, M., Årdal, C. and Jahre, M., 2024. Availability of essential, generic medicines before and during COVID-19 at selected public pharmaceutical supply agencies in Ethiopia: a comparative cross-sectional study | BMJ open | To assess the availability of essential, generic medicines for chronic diseases at public pharmaceutical supply agencies in Ethiopia. | Comparative cross-sectional study | public supply agency hub managers, warehouse managers and forecasting officers | January 14, 2024 | - The COVID-19 pandemic worsened the availability of essential chronic medicines, including higher rates of stock outs and unit price hikes for some products - The COVID-19 pandemic has aggravated already-existing medicine availability issues. - The mean availability of the selected basket medicines was reduced from 67.4% to 43.3% and the overall average line-item fill rate for the selected products was dropped from 78% to 49% during the pandemic. |
| 1. Ejeta, F., Feyisa, D., Kebede, O., Mechessa, D.F., Zewudie, A., Mamo, Y., Regasa, T. and Abebe, L., 2021. Logistics Management of Covid-19 Personal Protective Equipment and its Challenges at Public Hospitals of Southwest Ethiopia: An Integrated Quantitative and Qualitative Study | International Journal of Medical Research and Health Sciences | To assess the  logistics management of COVID-19 PPEs and its challenges at public hospitals of Benchi-sheko, Kafa, and Sheka  zones, southwest Ethiopia | A hospital-based descriptive cross-sectional  Study | 4 hospitals found in southwest Ethiopia | August 2021 | - All assessed hospitals had an insufficient quantity of personal protective equipment and poorly managing the inventory of available equipment - All hospitals were not used logistics   record-keeping tools (bin cards) for PPEs |
| 1. Dwindling medical supplies in northern Ethiopia prevents health workers from aiding those in need, Report of ICRC | Google | N/A | N/A | N/A | January 18, 2022 | - Some hospitals in Amhara have closed due to lack of medicines - In Tigray, single-use items such as gloves, surgical materials and even chest drains are being washed and reused, increasing the risk of infections - Patients are receiving expired medications, oxygen plants are not working anymore, and some health facilities cannot provide routine vaccines |
| 1. Adilo, T.M., Endale, S.Z., Demie, T.G. and Dinka, T.G., 2022. The impact of COVID-19 on supplies of routine childhood immunization in Oromia regional state, Ethiopia: A mixed method study. | Risk Management and Healthcare Policy journal | To assess the impact of COVID-19 on the availability of supplies of routine childhood immunization in the Oromia Regional State in Ethiopia. | facility-based mixed-methods | 450 health-care workers, 60 key informant interviews | Dec. 13, 2022 | - Corona Virus Disease −19 significantly disrupted the availability of supplies of childhood immunization in the Oromia region. - The most disrupted vaccines and related supplies were BCG, OPV, IPV, PENTA, facemasks, and hand gloves. |
| 1. Deressa, W., Worku, A., Abebe, W., Gizaw, M. and Amogne, W., 2021. Availability and use of personal protective equipment and satisfaction of healthcare professionals during COVID-19 pandemic in Addis Ababa, Ethiopia. | Archives of public health | To assess the availability and use of PPE, and satisfaction of HCPs with PPE in six public hospitals in Addis Ababa, Ethiopia. | Cross-sectional study | 1,134 HCPs | August 17, 2021 | - The majority (77%) of the HCPs reported that their hospital did not have adequate PPE - Almost 72% of the respondents were dissatisfied with the availability and use of PPE in their hospital. |
| **Health information systems (1)** | | | | | | |
| 1. Sasie, S.D., Van Zuylen, P., Ayano, G., Aragaw, F.M. and Spigt, M., 2024. Information sharing across institutions: Practices and barriers during public health emergencies in Ethiopia. | International Journal of Medical Informatics | To identify opportunities and challenges influencing practices to strengthen the national public health emergency response system. | mixed-methods study | 169 stakeholders and 23 in-depth interviews | June 2024 | - limited functionality of digital health systems - Incompatible data formats financial constraints and socio-cultural barriers constrain current practices in Ethiopia. |
| **Health workforce (15)** | | | | | | |
| 1. Mitike G, Nigatu F, Wolka E, Defar A, Tessema M, Nigussie T. Health system response to COVID-19 among primary health care units in Ethiopia: A qualitative study | PLoS One. | To explored how sub-national primary  health care units and coordinating bodies in Ethiopia responded to COVID-19 during the  first 6 months of pandemic | A qualitative study (phenomenological design) | 59 key  informants | 2023 Feb | - Regional, zonal, and district administrators formed multisectoral COVID-19 committees for planning/monitoring. - Health leaders reassigned staff, blocked leave, hired contract workers, and trained rapid response teams, lab techs, and community health workers. - Protective equipment and equipment were critically lacking initially; efforts to procure more were insufficient. - Awareness campaigns used media and door-to-door outreach. Rapid teams handled testing/tracing; mild cases shifted to home isolation due to limited facilities. - Essential services were disrupted, multisectoral coordination declined over time. |
| 1. Zewudie, A., Regasa, T., Kebede, O., Abebe, L., Feyissa, D., Ejata, F., … Mamo, Y. (2021). Healthcare Professionals’ Willingness and Preparedness to Work During COVID-19 in Selected Hospitals of Southwest Ethiopia. | Risk Management and Healthcare Policy | To identify barriers of willingness to work during epidemics outbreak and preparedness of healthcare professionals | Facility-based cross-sectional study | 407 healthcare professionals | 2021 | - Low healthcare professionals’ willingness and preparedness to work during COVID-19. - Use of Telemedicine, provision of personal protective equipment, increase hospital’s safety - Assigning staff who have experience of more than ten years in the risky wards of the hospitals decrease staffs absentee and increase in the provision of continuous service |
| 1. Firew T, Gebreyesus A, Woldeyohannes L, Ebrahim F, Patel S: Human resources for emergency care systems in Ethiopia: Challenges and triumphs. | African Journal of Emergency Medicine | To analyze the Challenges and triumphs of human resources for emergency care systems in Ethiopia |  |  | 2020 | - No sufficient specialized training of emergency medicine physicians at all hospitals - Lack of support from other specialties and timely emergency care has not been met for many reasons. |
| 1. Khatri, R.B., Endalamaw, A., Erku, D., Wolka, E., Nigatu, F., Zewdie, A. and Assefa, Y., 2023. Preparedness, impacts, and responses of public health emergencies towards health security: | Archives of public health | To synthesize available evidence on PHEs, their preparedness, impacts, and responses | Qualitative scoping review | 64 studies | 2023 | - Health systems of many low- and middle-income countries had inadequate preparedness to absorb the shocks of PHEs, limited surveillance, and monitoring of risks. - Health systems have been overburdened with interrupted health services, increased need for health services, poor health resilience, and health inequities. |
| 1. Gebrekidan AY, Enaro EY, Azeze G, Adella GA, Kassie GA, Haile KE, Asgedom YS: Turnover intention among healthcare workers in Ethiopia | BMJ Open | To determine the pooled prevalence of turnover intention among healthcare workers in Ethiopia. | A systematic review and meta-analysis | 29 cross-sectional studies with 9422 participants | 2023 | - High prevalence of turnover intention among healthcare workers in Ethiopia. |
| 1. Mekonnen Mihretie T, Kibret Abebe G, Mulugeta H, Tarekegn Kassaw A, Wondmagegn Alamaw A, Adugna B, Shenkute Ergetie F, Birara Zemariam A: Turnover intention and associated factors among nurses working at governmental hospitals in Bahir Dar city at the time of war, northwest Ethiopia, 2022. | International Journal of Africa Nursing Sciences | To assess Turn over intention of nurses | A cross-sectional study | 385 nurses | 2024 | - High prevalence of nurse turnover intention in Bahir Dar governmental hospitals - Satisfaction with their job significantly contribute for high nurses' turnover intention, |
| 1. Gesesew H, Berhane K, Siraj ES, Siraj D, Gebregziabher M, Gebre YG, Gebreslassie SA, Amdes F, Tesema AG, Siraj A et al: The impact of war on the health system of the Tigray region in Ethiopia: | BMJ Glob Health | Assessment of the health system before and during the war | An assessment | NA | 2021 | - Significant attrition of health workers, reduction in maternal and child health services and increase in rates of malnutrition, burden of infectious and non-infectious illness and gender-based violence. - significant infrastructural damage, and enormous consequences on the physical, mental and psychosocial health of millions of people. |
| 1. Nicolai, S., Diwakar, V., Khan, A., Mansour-Ille, D. and Anderson, A., 2020. Strengthening coordinated education planning and response in crisis contexts. Overseas Development Institute. | Overseas Development Institute | To understand the  formal coordination for education in emergencies  and protracted crises | Synthesis Report | NA | 2020 | - Many contexts have a lack of coordination leadership capacity, as a result of there being no coordination staff, of the staff not having the right profile, or there being little to no training on humanitarian programming. - The focus on and resourcing of information management and data collection appears relatively weak as part of education coordination in crisis contexts when the potential added value is considered. |
| 1. Etafa, W., Gadisa, G., Jabessa, S. and Takele, T., 2021. Healthcare workers’ compliance and its potential determinants to prevent COVID-19 in public hospitals in Western Ethiopia. | BMC infectious diseases | To assess health workers’ compliance with measures to prevent COVID-19, in public hospitals of Western Ethiopia. | cross-sectional survey | 422 healthcare workers | 19 May 2021 | - poor compliance of health workers with COVID-19 preventive measures - Providing information and refreshing training is important to improve the level of healthcare workers’ adherence with COVID-19 prevention |
| 1. Daba, C., Atamo, A., Weldehanna, D.G., Oli, A., Debela, S.A., Luke, A.O. and Gebrehiwot, M., 2023. Infection prevention and control compliance of healthcare workers towards COVID-19 in conflict-affected public hospitals of Ethiopia. | BMJ open. | To assess compliance with COVID-19 IPC measures among healthcare providers in Ethiopian governmental hospitals affected by conflict. | cross-sectional study | 325 healthcare workers | November 2023 | - High non-compliance with COVID-19 IPC. - Providing regular training on COVID-19 IPC, continuous piped water and personal protective facilities for healthcare workers may increase the non-compliance |
| 1. Kassahun, C.W., Abate, A.T., Tezera, Z.B., Beshah, D.T., Agegnehu, C.D., Getnet, M.A., Abate, H.K., Yazew, B.G. and Alemu, M.T., 2022. Working environment of nurses in public referral hospitals of West Amhara, Ethiopia | BMC nursing | To assess the working environment of nurses in Public Referral Hospitals in Public Referral Hospitals of West Amhara Regional State, Ethiopia | Institution based cross-sectional study | 423 nurses | 2022 | - More than half of nurses reported that their working environment was not healthy to appropriate practice |
| 1. SENDEKIE, T.Y., Temam, G., vas Roosmalen, J., Stekelenburg, J., Kim, Y.M., Shawula, S., Woldemariam, D. and Yilma, E., 2020. Satisfaction and turnover intention of physicians and public health officers in government health facilities: a national cross-sectional study. | Ethiopian Medical Journal | To assess job satisfaction and turnover intention of physicians and public health officers in government health facilities. | A national cross-sectional study | 375 physicians and 127 health officers | 2020 | - The level of dissatisfaction and turnover intention among physicians and health officers is considerable and driven largely from poor leadership & management, salary, and living conditions. |
| 1. Gebregziabher, D., Berhanie, E., Berihu, H., Belstie, A. and Teklay, G., 2020. The relationship between job satisfaction and turnover intention among nurses in Axum comprehensive and specialized hospital Tigray, Ethiopia. | BMC nursing | To assess the relationship between job satisfaction and turnover intention among nurses in Axum Comprehensive and Specialized Hospital Tigray, Ethiopia. | Institution based cross-sectional study | 148 nurses | 2020 | - The overall turnover intention of nurses was found to be high and significantly associated with dissatisfaction on autonomy, and training opportunity. |
| 1. Ethiopia: Army Attacks Health Care in Amhara Conflict | Human Rights Watch |  | News Release |  | 2024 | - Ethiopian security forces have committed widespread attacks amounting to war crimes against medical professionals, patients, and health facilities in the Amhara region. |
| 1. Ethiopia: Healthcare crisis in Oromia exacerbated by massive displacement | International Committee of the Red Cross |  | News release |  | 22-06-2023 | - The ongoing violence in the Oromia region of Ethiopia has taken a toll on critical infrastructure. Health facilities and water systems have been seriously affected. - In Begi, a district of 100,000 inhabitants, nearly all 42 existing health posts have been looted or damaged. Patients with life-threatening medical conditions cannot receive urgent care because health facilities are no longer functioning. |
| **Leadership and governance (5)** | | | | | | |
| 1. Rawat, A., Karlstrom, J., Ameha, A., Oulare, M., Omer, M.D., Desta, H.H., Bahuguna, S., Hsu, K., Miller, N.P., Bati, G.T. and Rasanathan, K., 2022. The contribution of community health systems to resilience: Case study of the response to the drought in Ethiopia. | Journal of Global Health | To identify salient factors that can improve health system resilience by exploring the successes and challenges experienced by a community-based health system during the drought response. | Qualitative (FGDs and KIIs) | 94 participants (71 from the communities and 23 from other levels). | 2022 | - Lack of international funding and Lack of adaptation due to long term vision is identified as challenge - Flexible human resources and coordination between facilities and communities and preparedness reduces outbreaks |
| 1. Nigussie, H., 2021. The coronavirus intervention in Ethiopia and the challenges for implementation. | Frontiers in Communication | To examine the COVID-19 intervention and the challenges for implementation in Mekelle, Tigray, northern Ethiopia. | Case study | 35 individual interviews, 15 hours of personal observation and four editorial policy documents | 2021 | - Majority of the population in Mekelle are aware of the pandemic and practiced hand washing with soap and water. - Limited physical distancing in religious institutions, market places, and coffee houses |
| 1. Zikargae, M.H., 2020. COVID-19 in Ethiopia: assessment of how the Ethiopian government has executed administrative actions and managed risk communications and community engagement. | Risk management and healthcare policy | Investigates all government and stakeholders’ effects in line with identifying the current status found in Ethiopia. | Qualitative | NA | 2020 | - The communication strategy and state of emergency are in place to reduce the prospective risks of COVID-19. - KAP’s practices are inadequate to fight and minimize the impacts of the pandemic. |
| 1. Assefa, Y., Woldeyohannes, S., Cullerton, K., Gilks, C.F., Reid, S. and Van Damme, W., 2022. Attributes of national governance for an effective response to public health emergencies: Lessons from the response to the COVID-19 pandemic. | Journal of Global Health | To identify the key attributes of governance in response to the COVID-19 pandemic and gain lessons for an effective response to public health emergencies | mixed-methods design |  | July 6, 2022 | - the current governance frameworks and measures are inadequate for understanding national governance of the COVID-19 epidemic |
| 1. Debela, B.K., 2020. The COVID-19 pandemic and the Ethiopian public administration: responses and challenges. Good public governance in a global pandemic, 113. | Good Public Governance in a Global Pandemic (book chapter) | To explore how the Ethiopian public  administration is responding to the virus | A narrative sythesis |  | 2020 | Identified challenges are:   - Ethiopian public administration is weak - dysfunctional distrust among political parties - lack of adequate inter-sectorial cooperation and coordination - deep-seated top-down planning and hierarchical - politico-administrative culture |
| **Healthcare Financing (5)** | | | | | | |
| 1. Debie, A., Khatri, R.B. and Assefa, Y., 2022. Contributions and challenges of healthcare financing towards universal health coverage in Ethiopia: a narrative evidence synthesis. | BMC health services research | To map the contributions, successes and challenges of HCF initiatives in Ethiopia. | A narrative evidence synthesis | 52 articles | 2022 | - Ethiopia's HCF initiatives have significant contributions to healthcare infrastructures, medical supplies, diagnostic capacity, drugs, financial-risk protection, and healthcare services. - Poor access to equitable quality healthcare services was associated with low healthcare funding and high OOP payments. |
| 1. Federal Ministry of Health, Annual Health Sector Performance Report | EMOH | This annual performance  report indicates the progress made in the first two years of the HSTP-II  period for selected core indicators of HSTP-II | Report |  | 2021/22 | - The conflict in the   northern and some other parts of the country, drought, flood and the continued COVID-19 pandemic affected service provision in some parts  of the country but the sector was able to overcome the effects to register major achievements in terms of improving access, utilization and health  outcomes. |
| 1. Priority humanitarian response and critical funding gaps |  | This document identifies priority activities for the coming three  months to enable the response in both drought and non-drought  affected areas in Ethiopia | An addendum to the 2024 Humanitarian Response Plan |  | February 2024 | - The CCCM cluster response has been challenged by the lack of funding that pushed some partners to stop their activities. Because of the funding limitation CCCM partners have been able to reach only 21% of sites, while living conditions in camps or camp-like settings continue to deteriorate, exacerbating the hardships faced by the displaced persons. In addition, an increasing number of Internally Displaced Persons (IDPs) are residing with host communities. |
| 1. Mumin AA, Oladeji O, Yohannes A: Factors Associated with Utilization of Donor Funds in Somali Region of Ethiopia. | Journal of Economics, Management and Trade | The study investigated the factors that influence the use of donor funds | cross-sectional survey | 79 key staff of eleven development partners and  United Nation Agencies | 2021 | - lower utilization rate of donor funds across all the sectors in the region compared to domestic funds with utilization rate for domestic resources ranged from 93%-100% compared with 43% utilization rate for donor funds. |
| 1. Wondimu, W. and Girma, B., 2020. Challenges and silver linings of COVID-19 in Ethiopia–short review. | Journal of Multidisciplinary Healthcare | To discuss the challenges of COVID-19 in Ethiopia in terms of health, economy, and social life, and the silver linings in terms of research opportunities, technological transformation, reading culture, and family discussion | Literature review |  | Sep. 16, 2020 | - COVID-19 has negative impacts on the health system, economy, and social life in Ethiopia, |
